# A preliminary study of osteochondral regeneration using a scaffold-free three-dimensional construct of porcine adipose tissue-derived mesenchymal stem cells

**DOI:** 10.1186/s13018-015-0173-0

**Published:** 2015-03-18

**Authors:** Daiki Murata, Satoshi Tokunaga, Tadashi Tamura, Hiroaki Kawaguchi, Noriaki Miyoshi, Makoto Fujiki, Koichi Nakayama, Kazuhiro Misumi

**Affiliations:** Veterinary Surgery, Department of Veterinary Clinical Science, Joint Faculty of Veterinary Medicine, Kagoshima University, 21-24 Korimoto 1-chome, Kagoshima, 890-0065 Japan; Veterinary Teaching Hospital, Joint Faculty of Veterinary Medicine, Kagoshima University, 21-24 Korimoto 1-chome, Kagoshima, 890-0065 Japan; Cyfuse Biomedical K.K., 1-1 Maidashi 3-chome, Higashi-ku, Fukuoka 812-8582 Japan; Veterinary Pathology, Department of Pathological and Preventive Sciences, Joint Faculty of Veterinary Medicine, Kagoshima University, 21-24 Korimoto 1-chome, Kagoshima, 890-0065 Japan; Department of Advanced Technology Fusion, Graduate School of Science and Engineering, Saga University, Honjyo 1-chome, Honjyo-cho, Saga 840-8502 Japan

**Keywords:** Regeneration, Cartilage, Bone, Scaffold-free, Three-dimensional construct, Stem cell, Adipose tissue, Computed tomography, Histopathology, Porcine

## Abstract

**Background:**

Osteoarthritis (OA) is a major joint disease in humans and many other animals. Consequently, medical countermeasures for OA have been investigated diligently. This study was designed to examine the regeneration of articular cartilage and subchondral bone using three-dimensional (3D) constructs of adipose tissue-derived mesenchymal stem cells (AT-MSCs).

**Methods:**

AT-MSCs were isolated and expanded until required for genetical and immunological analysis and construct creation. A construct consisting of about 760 spheroids that each contained 5.0 × 10^4^ autologous AT-MSCs was implanted into an osteochondral defect (diameter: 4 mm; depth: 6 mm) created in the femoral trochlear groove of two adult microminipigs. After implantation, the defects were monitored by computed tomography every month for 6 months in animal no. 1 and 12 months in animal no. 2.

**Results:**

AT-MSCs were confirmed to express the premature genes and to be positive for CD90 and CD105 and negative for CD34 and CD45. Under specific nutrient conditions, the AT-MSCs differentiated into osteogenic, chondrogenic, and adipogenic lineages, as evidenced by the expressions of related marker genes and the production of appropriate matrix molecules. A radiopaque area emerged from the boundary between the bone and the implant and increased more steadily upward and inward for the implants in both animal no. 1 and animal no. 2. The histopathology of the implants after 6 months revealed active endochondral ossification underneath the plump fibrocartilage in animal no. 1. The histopathology after 12 months in animal no. 2 showed not only that the diminishing fibrocartilage was as thick as the surrounding normal cartilage but also that massive subchondral bone was present.

**Conclusions:**

The present results suggest that implantation of a scaffold-free 3D construct of AT-MSCs into an osteochondral defect may induce regeneration of the original structure of the cartilage and subchondral bone over the course of 1 year, although more experimental cases are needed.

**Electronic supplementary material:**

The online version of this article (doi:10.1186/s13018-015-0173-0) contains supplementary material, which is available to authorized users.

## Background

Osteoarthritis (OA) is a major joint disease contributing to midlife and geriatric locomotor dysfunction, and the associated disability can decrease quality of life in humans [1]. OA slowly progresses not only as a result of traumatic injuries to joint structures [2] but also through many exacerbating factors such as age, sex, body mass index, occupation, bone shape, and genetic factors regulating proteolytic enzymes [3-5]. In advanced OA, cartilage degeneration and subchondral bone sclerosis may be worsened by the usual mechanical load of daily activities [6], and therefore surgical strategies to reconstruct both the bone and cartilage have been investigated to restore joint structure and function [7]. A particular issue of interest in recent studies has been the complete regeneration of hyaline cartilage covering the subchondral bone.

Although a clinical study of osteochondral autografts from non-load-bearing sites implanted into deteriorated sites showed favorable outcomes following surgery (clinical improvement in 79%–94% of OA patients) [8], a loss of clinically sound cartilage at the donor sites is associated with autologous osteochondral transfer [9]. Studies on surgical procedures using a combination of artificial bone and autologous chondrocytes seeded into a collagen scaffold have also shown favorable restoration of cartilage [10,11]. However, some studies have suggested associated problems such as isolation of few chondrocytes from a small piece of normal cartilage [10] and dedifferentiation of chondrocytes during passages in culture [12]. To solve these problems, stem cells have recently received attention in a study [13].

Stem cells are defined as immature cells that have the ability for self-renewal and the potential for multilineage differentiation into specific cells. Mesenchymal stem cells (MSCs) derived from bone marrow (BM) and adipose tissue (AT) have mostly been used to demonstrate differentiation into bone and cartilage in vitro [14,15]. BM-derived MSCs (BM-MSCs) appear to have some disadvantages including decreased numbers and deterioration of the cells depending on senescence and natural transformation caused by genomic instability [16]. Previous experiments have shown age-related decreases in the yield rate, growth rate, and differentiation potential of BM-MSCs in rats and humans [17,18]. On the other hand, the advantages of AT-derived MSCs (AT-MSCs) are that abundant cells can be isolated from AT and their cellular proliferation rate may be higher in mature animals [19]. Furthermore, given that obesity is undesirable in OA patients, the regenerative strategy for bone and cartilage using unwanted AT could be reasonable and acceptable by many OA patients. It has been reported that AT-MSCs hardly differentiate into chondrocytes [20]. However, a recent study using rabbits demonstrated the regeneration of bone and cartilage after implantation of scaffold-free three-dimensional (3D) constructs of AT-MSCs into osteochondral defects [21]. This report also contains a novel strategy for scaffold-free cell implantation.

Previous studies indicated that scaffolds composed of materials such as collagen and hyaluronic acid could be useful for promoting cell adhesion, proliferation, and chondrogenic differentiation [22,23], and bone regeneration using AT-MSCs seeded into hydroxyapatite has also been investigated [24]. However, artificial materials may induce xenobiotic reactions through immune reactions in the tissue [25].

In many previous studies, bone and cartilage regeneration through various cell therapies has been evaluated in the knee joint of rabbits [26-30]. However, to obtain meaningful results that are appropriate for extrapolating bone and cartilage regeneration to human OA, we expect that pigs will provide a more appropriate animal model than rabbits. Microminipigs (MMPigs) have similar behavior patterns to human daily life, as they spend time standing and walking in the daytime and sleeping at night [31,32]. In contrast, rabbits usually sit in cages. This study was designed to evaluate the regeneration of articular cartilage and subchondral bone using 3D constructs of autologous AT-MSCs in MMPigs.

## Materials and methods

### Animals

Two MMPigs (Fuji Micra, Shizuoka, Japan), designated animal no. 1 (male) and animal no. 2 (female), were used in this study. Their body weights and ages were 13.8 kg and 25 months, and 14.6 kg and 23 months, respectively. All procedures in this study were approved by the Animal Care and Use Committee of Kagoshima University (Approval No. A11037). Ten to fifteen grams of cervical AT per animal was aseptically obtained under general anesthesia.

### Isolation and expansion of AT-MSCs

The AT samples were minced and digested for 90 min in phosphate-buffered saline (PBS) containing 0.1% collagenase (Collagenase Type I; Worthington Biochemical, Lakewood, NJ). The digested cell suspensions were filtered through a 70-μm-pore-diameter membrane (Cell Strainer; BD, Franklin Lakes, NJ) and centrifuged at 160 × *g* for 5 min at room temperature. After decanting the supernatant, the pellet was resuspended with PBS and centrifuged. The supernatant was removed, and the pellet was resuspended and plated on a 150-cm^2^ culture dish (Tissue Culture Dish φ 150; TPP, Trasadingen, Switzerland) in complete culture medium (CCM): Dulbecco’s modified Eagle’s medium (DMEM; Life Technologies, Carlsbad, CA) containing 10% fetal bovine serum (FBS; Thermo Fisher Scientific, Waltham, MA) and 1% antibiotic-antifungal preparation (100 U/ml penicillin G, 100 μg/ml streptomycin, 0.25 μg/ml amphotericin B; Antibiotic-Antimycotic; Life Technologies). Following incubation at 37 °C under 5% CO_2_ for 7 days, the cells adhering to the bottom of the dish were washed with PBS and cultured in CCM. The medium was changed on day 7 at passage 0. At day 10, the cells were harvested with 0.25% trypsin and 1 mM EDTA (Trypsin-EDTA; Life Technologies) diluted by adding five volumes of PBS and centrifuged. After decanting the supernatant, the pellet was rinsed with CCM, and the cells were replated at 5 × 10^5^ cells per 150-cm^2^ dish and cultured for 6 days. The medium was changed every 3 days for 6 days during passage 1. This serial process of passaging was repeated until the cells were required for analysis and construct creation. The cells were used for creating the constructs at passage 4. Immunological surface markers and multipotency of the cells were analyzed at passage 5.

### Genetic and molecular specificity of AT-MSCs

Ten thousand cells were used to analyze the specific gene expressions in MSCs. Total RNA from the cells was prepared with an RNA isolation kit (MirVana miRNA Isolation Kit; Life Technologies), according to the manufacturer’s instructions. The isolated RNA was converted to cDNA and amplified with a TAKARA RT-PCR system (PCR Thermal Cycler MP; Takara Bio, Otsu, Japan) and RT-PCR kit (ReverTra Dash; Toyobo, Osaka, Japan). Specific PCR primers were used to amplify octamer-binding transcription factor 4 (OCT-4), sex-determining region Y box 2 (SOX-2), Krüppel-like factor 4 (KLF-4), cellular myelocytomatosis oncogene (C-MYC), and homeobox protein NANOG (NANOG) as premature marker genes. The conditions and expected sizes of the products are summarized in Table 1. Ten thousand cells were resuspended in 500 μl of staining buffer (SB; PBS containing 1% FBS) and incubated for 30 min at 4 °C with 20 μg/ml FITC-conjugated antibodies against CD34 (BD), CD45 (BD), CD90 (BD), or CD105 (Abcam, Cambridge, UK). Non-specific FITC-conjugated mouse immunoglobulin G1κ (BD) was used as a negative control. The characteristics of the antibodies are listed in Table 2. The FITC-labeled cells were washed with SB and resuspended in 500 μl of SB for fluorescence-activated cell sorting (FACS) analysis. Cell fluorescence was evaluated as a strong shift in the mean fluorescence intensity (MFI) on flow cytometry using a FACSAria II instrument (BD). The data were analyzed using FACSDiva software (BD).

**Table 1:** **List of PCR primers**

**Marker**	**Gene**	**Sequence (forward/reverse)**	**Ann. temp. (°C)**	**Fragment (bp)**
Premature	OCT-4	5′-GTCGCCAGAAGGGCAAAC-3′	57.0	157
		5′-CAGGGTGGTGAAGTGAGGG-3′		
	SOX-2	5′-CCCTGCAGTACAACTCCATGAC-3′	59.0	85
		5′-GGTGCCCTGCTGCGAGTA-3′		
	KLF-4	5′-CGGCAAAACCTACACGAAGAGT-3′	59.0	119
		5′-AGTTCATCTGAGCGGGCAAAT-3′		
	NANOG	5′-CTTATTCAGGACAGCCCTGATTCTTC-3′	59.0	613
		5′-AAGACGGCCTCCAAATCACTG-3′		
	C-MYC	5′-GGATTCCGCCTCGTT-3′	55.1	184
		5′-TCTCCAAGCATCACTCG-3′		
Osteogenic	ALP	5′-ATGAGCTCAACCGGAACAA-3′	56.0	131
		5′-GTGCCCATGGTCAATCCT-3′		
	OC	5′-TCAACCCCGACTGCGACGAG-3′	68.0	204
		5′-TTGGAGCAGCTGGGATGATGG-3′		
	ON	5′-TCCGGATCTTTCCTTTGCTTTCTA-3′	57.5	187
		5′-CCTTCACATCGTGGCAAGAGTTTG-3′		
Chondrogenic	SOX-9	5′-CCGGTGCGCGTCAAC-3′	57.5	119
		5′-TGCAGGTGCGGGTACTGAT-3′		
	AGG	5′-TTCCCTGAGGCCGAGAAC-3′	65.5	194
		5′-GGGCGGTAATGGAACACAAC-3′		
Adipogenic	PPAR-γ2	5′-GCGCCCTGGCAAAGCACT-3′	59.8	238
		5′-TCCACGGAGCGAAACTGACAC-3′		
	AP2	5′-GGCCAAACCCAACCTGA-3′	59.8	167
		5′-GGGCGCCTCCATCTAAG-3′		
Housekeeping	GAPDH	5′-ACCACAGTCCATGCCATCAC-3′	60.0	450
		5′-TCCACCACCCTGTTGCTGTA-3′		

*OCT-4* octamer-binding transcription factor 4, *SOX-2* sex-determining region Y box 2; *KLF-4* Krüppel-like factor 4, *NANOG*, homeobox protein NANOG, *C-MYC*, cellular myelocytomatosis oncogene, *ALP* alkaline phosphatase, *OC* osteocalcin, *ON* osteonectin, *SOX-9* sex-determining region Y-box 9, *AGG* aggrecan, *PPAR-γ2* peroxisome proliferator-activated receptor γ2, *AP2* adipocyte fatty acid-binding protein 2, *GAPDH* glyceraldehyde-3-phosphate dehydrogenase.

**Table 2:** **List of antibodies**

**Antibody**	**Company**	**Clone**	**Epitope**	**Dilution**
CD34	BD	581	O-glycosylated transmembrane glycoprotein	1:5
CD45	BD	2D1	T200 family	1:2.5
CD90	BD	5E10	N-glycosylated GPI-linked membrane glycoprotein	1:10
CD105	Abcam	MEM229	Disulfide-linked glycoprotein homodimer	1:20
Isotype	BD	MOPC-21	(Not confirmed)	1:10

### Tri-lineage analysis

To investigate osteogenic differentiation, the AT-MSCs were placed in six-well plates (6 Well Plate-N; Nest Biotech, Wuxi, China) in CCM at an initial density of 5,000 cells/cm^2^. After 24 h of incubation, the medium was replaced with osteogenic induction medium (Differentiation Basal Medium—Osteogenic; Lonza, Walkersville, MD), supplemented with 100 μM ascorbic acid, 10 mM β-glycerophosphate, and 1 μM dexamethasone, for 2 weeks. To investigate chondrogenic differentiation, AT-MSCs (5 × 10^5^) were resuspended in a 15-ml culture tube (SuperClear centrifuge tubes; Labcon, Petaluma, CA) in 500 μl of chondrogenic induction medium (Differentiation Basal Medium—Chondrogenic, Lonza), supplemented with 4.5 g/l d-glucose, 350 μM l-proline, 100 nM dexamethasone, and 0.02 g/l transforming growth factor beta 3. Chondrogenic differentiation was induced in pellet cultures for 2 weeks. Adipogenic differentiation began when AT-MSCs reached a density of 5,000 cells/cm^2^ in six-well plates in CCM. Following a 24-h preincubation, the medium was replaced with Adipogenic Induction Medium (Lonza), supplemented with 4.5 g/l d-glucose, 100 μM indomethacin, 10 μg/ml insulin, 0.5 mM 3-isobutyl-1-methylxanthine, and 1 μM dexamethasone, for 3 days for induction of specific genes and molecules.

The PCR primers and conditions, and the expected sizes of the products are summarized in Table 1. The osteogenic marker genes were osteocalcin (OC), osteonectin (ON), and alkaline phosphatase (ALP). The chondrogenic marker genes were sex-determining region Y-box 9 (SOX-9) and aggrecan (AGG). The adipogenic marker genes were adipocyte fatty acid-binding protein 2 (AP2) and peroxisome proliferator-activated receptor γ2 (PPAR-γ2). The reaction products were electrophoresed in a 2% agarose gel (Agarose XP; Wako Pure Chemical Industries, Osaka, Japan), and the expressions of the specific genes were determined based on the expected sizes of the bands labeled with SYBR Green (Takara Bio).

Production of calcium apatite crystals in the osteogenic extracellular matrix was evaluated with alizarin red staining in the wells of culture plates. The chondrogenic cell pellets were fixed with 10% neutral buffered formalin (NBF), embedded in paraffin, and cut into 5-μm sections using a microsection instrument. The sections were stained with alcian blue to detect cartilage-specific proteoglycans. Adipocyte-specific intracellular lipids were stained with oil red O.

### Preparation and implantation of 3D constructs of AT-MSCs

**Figure 1:**
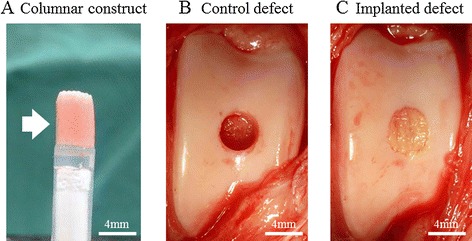
**Surgical procedure.** A columnar construct (4 mm in diameter and 6 mm in height) for the implantation **(A)**. A cylindrical osteochondral defect in each groove before implantation **(B)**. The construct composed of about 760 spheroids of AT-MSCs was autografted into the osteochondral defect in the right hind limb **(C)**. Nothing was implanted into the left limbs (control defects; **B**).

At least 4 × 10^7^ AT-MSCs were used to produce each autologous construct. The cells were inoculated into eight 96-well plates (Sumitomo Bakelite, Tokyo, Japan) with 5 × 10^4^ cells/well. After undisrupted incubation for 48 h, the cells formed spheroids with a diameter of about 700 μm in the bottom of the wells. About 760 spheroids were placed in a cylindrical mold and incubated in CCM until implantation (7 days). When the mold was carefully removed, a columnar construct of 4 mm in diameter and 6 mm in height appeared and was used for autologous implantation (Figure 1A). The general outline of this method of construction has already been reported [21,33].

The implant surgery was performed under general anesthesia using oxygen and isoflurane inhalation following premedication with sedatives and analgesics. Both femoropatellar joints were incised from the outside, and the femoral trochlear groove was exposed. Using a surgical trephine with an outer diameter of 4 mm, the articular cartilage and subchondral bone were drilled to a depth of 6 mm at the center of the groove. After removing a column of cartilage and bone, a cylindrical osteochondral defect was created in each groove (Figure 1B). A columnar construct (4 mm in diameter and 6 mm in height) composed of spheroids of AT-MSCs was autografted into the osteochondral defect in the right hind limb (Figure 1C), while no graft was implanted into the defect in the left limb (control defect, Figure 1B).

### Assessment of osteochondral defects

Postoperatively, the implants and osteochondral defects were followed up every month for 6 months in animal no. 1 and 12 months in animal no. 2 using computed tomography (CT) scans of both stifles. For assessment, longitudinal section images were obtained at the maximum diameter in lateral views of the cylindrical defect and the maximum diameters of the radiolucent area in the images were evaluated at 0.5-mm intervals between 0 and 9 mm.

Animal no. 1 was euthanized at 6 months after surgery, and animal no. 2 was euthanized at 12 months after surgery. The macroscopic findings were scored with the International Cartilage Repair Society (ICRS) gross grading scale (Table 3). Both distal femurs were fixed in 10% NBF for 1 week and then longitudinally sectioned parallel to the trochlear groove. The tissue was decalcified with formic acid for 1 week and embedded in paraffin. Serial sections (3-μm thickness) were placed on glass slides and evaluated by Masson’s trichrome staining, alcian blue staining, and immunohistochemistry using specific antibodies against collagen type II (Col-II; 1:100 dilution; Daiichi Fine Chemicals, Takaoka, Japan) and an Avidin-Biotin Enzyme Complex system (VECTASTAIN ABC Standard Kit; Vector Laboratories, Southfield, MI). The histopathologic findings were scored with the ICRS histological grading scale (Table 4).

**Table 3:** **ICRS gross grading scale**

**Feature**		**Score**	**Animal no. 1**		**Animal no. 2**	
			**Control site**	**Implanted site**	**Control site**	**Implanted site**
Coverage	>75% fill	4	2	3	4	4
	50%–75% fill	3				
	25%–50% fill	2				
	<25% full	1				
	No fill	0				
Neocartilage color	Normal	4	1	2	3	4
	25% yellow/brown	3				
	50% yellow/brown	2				
	75% yellow/brown	1				
	100% yellow/brown	0				
Defect margins	Invisible	4	1	2	3	3
	25% circumference visible	3				
	50% circumference visible	2				
	75% circumference visible	1				
	Entire circumference visible	0				
Surface	Smooth/level with normal	4	0	2	3	3
	Smooth but raised	3				
	Irregular 25%–50%	2				
	Irregular 50%–75%	1				
	Irregular >75%	0				
Average (0–4)			1.0	2.25	3.25	3.5

**Table 4:** **ICRS histological grading scale**

**Feature**		**Score**	**Animal no. 1**		**Animal no. 2**	
			**Control site**	**Implanted site**	**Control site**	**Implanted site**
Surface	Smooth/continuous	3	0	3	3	3
	Discontinuities/irregularity	0				
Matrix	Hyaline	3	0	1	1	2
	Mixture; hyaline/fibrocartilage	2				
	Fibrocartilage	1				
	Fibrous tissue	0				
Cell distribution	Columnar	3	0	0	0	2
	Mixed/columnar clusters	2				
	Clusters	1				
	Individual cells/disorganized	0				
Viability of cell population	Predominantly viable	3	3	3	3	3
	Partially viable	1				
	<10% viable	0				
Subchondral bone	Normal	3	1	2	0	2
	Increased remodeling	2				
	Bone necrosis/granulation tissue	1				
	Detached/fracture/callus at base	0				
Cartilage mineralization (calcified cartilage)	Normal	3	0	3	3	3
	Abnormal/inappropriate location	0				
Average (0–3)			0.67	2	1.67	2.5

## Results

### Genetic and molecular characteristics and tri-lineage potential of AT-MSCs

**Figure 2:**
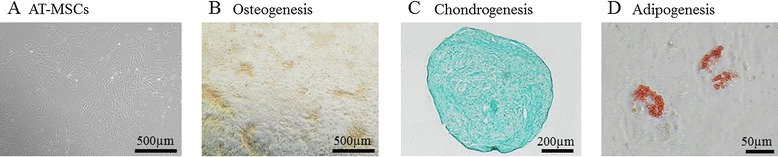
**Representative images of special staining and RT-PCR results of tri-lineage differentiation in AT-MSCs.** AT-MSCs adhering to the bottom of the culture dish were spindle-shaped **(A)**. Following 2 weeks of osteogenic induction, MSCs also showed characteristics of the stroma, including staining with alizarin red, indicating the presence of calcium apatite crystals **(B)**. Observation of the cell pellets that were induced by chondrogenic induction medium for 2 weeks showed a cartilage-like structure that was positively stained with alcian blue **(C)**. Adipogenic induction of the MSCs resulted in adipocyte-like flattened cells with small lipid vesicles that stained positively with oil red O **(D)**.

**Figure 3:**
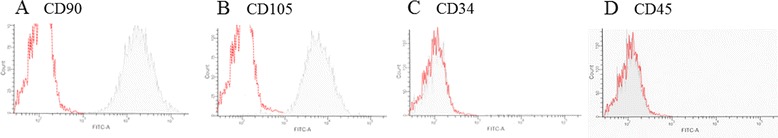
**Flow cytometry results of immunological markers in AT-MSCs.** A strong shift in MFI was detected with antibodies against CD90 **(A)** and CD105 **(B)**, whereas no signal reaction was detected with antibodies against CD34 **(C)** and CD45 **(D)**.

**Figure 4:**
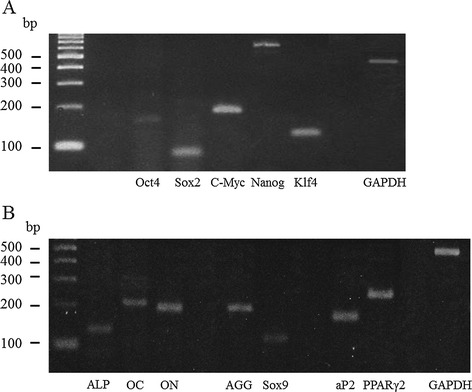
**RT-PCR results of gene expression in AT-MSCs.** Premature gene expression in AT-MSCs **(A)** and specific marker gene expression in AT-MSCs induced by tri-lineage differentiation medium **(B)** were confirmed.

Porcine AT-MSCs adhering to the bottom of the culture dish were spindle-shaped and proliferated well (Figure 2A), reaching over 1 × 10^6^ and 1 × 10^7^ cells at passage 3 and passage 4, respectively. A strong shift in MFI on flow cytometry was detected with antibodies against CD90 and CD105 (Figure 3A, B), while no signals were detected with antibodies against CD34 and CD45 (Figure 3C, D). The genetic markers of OCT-4, SOX-2, KLF-4, C-MYC, and NANOG were all positive (Figure 4A). Following osteogenic induction, AT-MSCs aggregated and contracted to form colonies (Figure 2B), and expressions of specific marker genes, including ALP, OC, and ON, were detected (Figure 4B). These cells also showed appropriate characteristics of the stroma, including staining with alizarin red, indicating the presence of calcium apatite crystals (Figure 2B). Reverse transcription PCR (RT-PCR) of AT-MSCs placed in chondrogenic induction medium revealed the expressions of marker genes, including SOX-9 and AGG (Figure 4B). Histological observation of the cell pellets showed a hyaline cartilage-like structure that was positively stained with alcian blue (Figure 2C). Adipogenic induction of the AT-MSCs resulted in adipocyte-like flattened cells with small lipid vesicles that were positively stained with oil red O (Figure 2D). RT-PCR revealed significant increases in adipogenic marker gene expressions such as AP2 and PPAR-γ2 (Figure 4B).

### CT images

**Figure 5:**
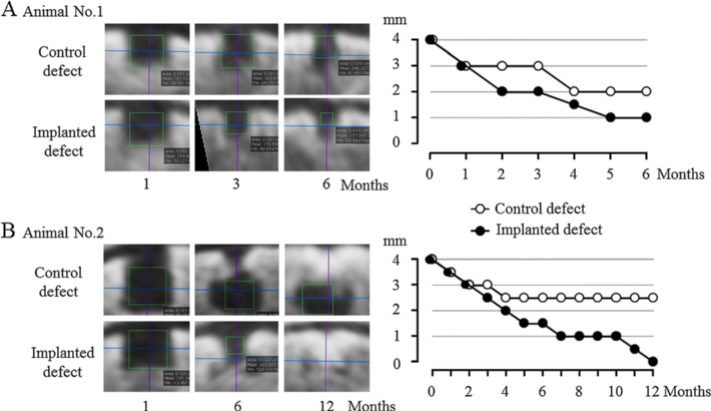
**CT assessment of osteochondral defects.** The upper image shows one cross section of the multiplanar reconstruction images 1, 3, and 6 months after the surgery in animal no. 1 **(A)**. The lower image shows one cross section of the multiplanar reconstruction images 1, 6, 12 months after the surgery in animal no. 2 **(B)**.

The reduction in the subchondral radiolucent area of the implanted site became more dramatic at 2 or 3 months after surgery compared with the control site in the both animals (Figure 5). CT images at 6 months after surgery for animal no. 1 are shown in Figure 5A. A radiopaque area emerged from the boundary between the bone and the implant and increased more steadily upward and inward for the implanted defect (the right femur) as time passed after surgery, compared with the control site. The radiolucent area of the implant diminished in a stepwise manner and then degraded to a diameter of 1 mm by 5 months after surgery. CT images at 12 months after surgery for animal no. 2 are shown in Figure 5B. A radiopaque area of the implant emerged in the same manner as in animal no. 1, gradually progressed, and then filled the entire osteochondral defect at 12 months after surgery. On the other hand, in the control site, a radiopaque area emerged in the shallow layer, but bone formation was not completed in the deep layer. The maximum diameter of the radiolucent area in the implanted site diminished in a stepwise manner and became 0 mm at 12 months after surgery. The control site remained at a diameter of 2.5 mm.

### Macroscopic appearance and histopathology of the osteochondral defects

**Figure 6:**
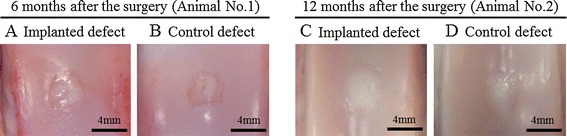
**Macroscopic findings of the surface of the implanted and control sites.** In animal no. 1, the surface of the implanted defect was covered with the abundant cartilaginous white tissues **(A)**, whereas the cartilaginous tissue was scarce and the surface was depressed in the control site **(B)**. In animal no. 2, the surface was more uniformly covered with abundant cartilaginous white tissues and the boundary to the surrounding normal cartilage was unclear in the implanted site **(C)**, comparing to those of the control site **(D)**.

Macroscopic examination of animal no. 1 revealed that the surface of the implanted defect was covered with abundant cartilaginous white tissue (Figure 6A), while cartilaginous tissue was scarce and the surface was depressed in the control site (Figure 6B). Similarly, in animal no. 2, the surface was quite uniformly covered with abundant cartilaginous white tissues and the boundary to the surrounding normal cartilage was unclear in the implanted site (Figure 6C), compared with the findings at the control site (Figure 6D). The average macroscopic scores for the implanted site were higher than those for the control site in animal no. 1, while the differences between the scores for the implanted site and the control site were decreased in animal no. 2 (Table 3).

**Figure 7:**
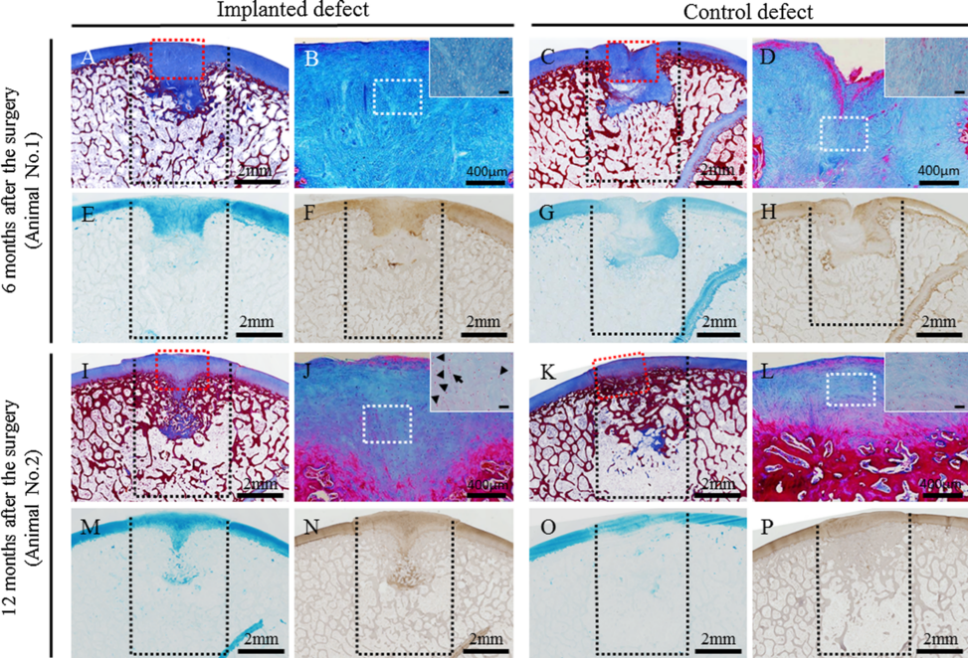
**Histopathology of osteochondral defects using Masson’s trichrome, alcian blue, and immunohistochemical staining of type II collagen.** In animal no. 1, the articular surface was smooth and fibrocartilage developed on the subchondral bone at the implanted site **(A, B, E, F)**, whereas the surface was irregular and fibrous tissue lay over the subchondral bone at the control site **(C, D, G, H)**. At the implanted site in animal no. 2, the subchondral bone was symmetrically reconstructed and was covered by matrix including hyaline cartilage, which was suggested by the clusters (arrowhead) and columnar clusters (arrow) of cells **(I, J, M, N)**. On the other hand, smooth and continuous surface was restored due to fibrocartilage formation, but subchondral bone was absent in the bottom half of the defect, at the control site in animal no. 2 **(K, L, O, P)**. Black dotted lines indicate the areas of osteochondral defects immediately after the surgery. Masson’s trichrome staining sections **(B, D, J, L)** were enlarged from red dotted square in the images **A**, **C**, **I**, and **K**, respectively. The insert images in sections **B**, **D**, **J**, and **L** were enlarged from white dotted square in images **B**, **D**, **J**, and **L**, respectively. The bars in the insert images indicate 50 μm.

Histopathological sections of animal no. 1 at 6 months after surgery showed that thickened fibrocartilage had developed over the subchondral bone that was regenerating in the implanted site (Figure 7A, B). The surface of the cartilage was smooth, and the boundary with the surrounding normal cartilage was obscure at the implanted site (Figure 7A). Meanwhile, the surface was collapsed and irregular at the control site (Figure 7C, D). The fibrocartilage showed more intense alcian blue staining and Col-II immunostaining at the implanted site (Figure 7E, F) compared with the control site (Figure 7G, H). In animal no. 2 at 12 months after surgery, partially thickened fibrocartilage was mounted on developed subchondral bone at the implanted site (Figure 7I, J). The surface of the cartilage was smooth, and the boundary with the surrounding normal cartilage was obscure, although small areas of endochondral ossification persisted at the center, and small amounts of AT had differentiated at the bottom part of the site (Figure 7I). Subchondral bone was symmetrically reconstructed in the defect and was covered by a mixed matrix of hyaline cartilage and fibrocartilage, in which clusters and columnar clusters of cells were observed (Figure 7J). In the control site, fibrocartilage had immediately covered the defect, but the subchondral ossification was poor (Figure 7K, L). The hyaline cartilage showed more intense and uniform alcian blue staining and Col-II immunostaining at the implanted site (Figure 7M, N) compared with the control site (Figure 7O, P). The averages of histologic scores for the implanted site were distinctly higher than those for the control site in both animals (Table 4).

## Discussion

Human AT-MSCs have been shown to be positive for CD90, which suppresses the cancerization of stem cells [34], and CD105, which is associated with cellular responses to blood vessel formation and TGF-β1 [34]. The porcine AT-derived and spindle-shaped cells adhering to the bottom of the culture dish in the present study were strongly positive for CD90 and CD105. CD34, which is involved in cell adhesion and is expressed in hematopoietic stem cells [34], and CD45, which activates T and B lymphocyte receptors in hematopoietic cells [34], were both negative in the porcine AT-derived cells. Because human hematopoietic cells, but not human MSCs, were positive for these molecules [35,36], the porcine AT-derived cells may not be contaminated with hematopoietic cells [37]. Genetic markers specific for human MSCs, such as SOX-2, OCT-4, NANOG [38], KLF-4, and C-MYC [39], were detected in the porcine cells by RT-PCR [40]. Moreover, the osteogenic, chondrogenic, and adipogenic potential of the cells was confirmed, and we therefore defined them as porcine AT-MSCs.

In accordance with a previously described procedure [21,33], we constructed scaffold-free 3D implants (diameter: 4 mm; height: 6 mm) composed of 760 spheroids each containing 5 × 10^4^ autologous AT-MSCs. The cross-sectional CT images obtained at 6 and 12 months after implantation in animal no. 1 and animal no. 2, respectively, may mirror the histology because the localization, size, and shape of the radiolucent and radiopaque areas entirely corresponded with those of the fibrocartilage and regenerated bone. To further discriminate between cartilaginous and fibrous tissues in the radiolucent area, magnetic resonance imaging should be used.

The higher average macroscopic scores may suggest better improvement in superficial features at the implanted site, compared with the control site (Table 3). However, the differences in the average scores between the control and implanted sites were lower in animal no. 2 (euthanized at 12 months after surgery) than in animal no. 1 (euthanized at 6 months after surgery). All four features in the ICRS gross grading scale system were improved at the implanted site compared with the control defect site in animal no. 1, whereas a difference in neocartilage color only was seen between the two sites in animal no. 2. The results in animal no. 2 were not consistent with a previous study using rabbits, in which a more degraded macroscopic appearance of the control defect (diameter: 4.8 mm; depth: 5 mm) was observed at 12 months after implantation [21]. Based on the results, we speculate that the superficial features may improve spontaneously from 6 to 12 months after surgery for this size of osteochondral defect (diameter: 4 mm; depth: 6 mm) in MMPigs. To discriminate the superficial features caused by spontaneous repair from those caused by MSC-based regeneration in this size of defect, further evaluation of the pathology at 6 months after surgery will be appropriate in MMPigs. Other studies are needed to determine methods for repairing osteochondral defects with larger diameters and depths, which could never repair by themselves (as shown in the Additional files 1 and 2).

We also obtained higher average histologic scores at the implanted sites in both animals, which may indicate desirable osteochondral recovery compared with the control site (Table 4). As summarized in Table 5, regarding the histological features in animal no. 1, a smooth and continuous surface was restored by thickened fibrocartilage at the implanted site, whereas the surface was collapsed and irregular at the control site. Fibrocartilage formation and endochondral ossification during the process of MSC-based regeneration were present at the implanted site, compared with fibrous granulation matrix and inadequate bone formation in the control defect. On the other hand, in animal no. 2, a smooth and continuous articular surface was restored through cartilage formation at both sites, but subchondral bone formation was distinctly more satisfactory at the implanted site than at the control site, in which the trabecular pattern was completely absent (bone was detached) in the bottom half of the defect. Subchondral bone was covered by a mixed matrix of hyaline cartilage and fibrocartilage at the implanted site, while fibrocartilage had immediately covered the defect at the control site. These findings were similar to data reported previously reported in rabbits [21] and may suggest transformation of fibrocartilage into hyaline cartilage during the process of MSC-based osteocartilage regeneration. Because neither hyaline cartilage nor cell clusters were seen in the implanted defect site in animal no. 1, transformation of fibrocartilage into hyaline cartilage may begin between 6 and 12 months after implantation. However, more time may be required to regenerate pure, high-quality hyaline cartilage as well as complete subchondral regeneration in the implanted defect.

**Table 5:** **Summary of histological features**

		**Animal no. 1**		**Animal no. 2**	
		**Control site**	**Implanted site**	**Control site**	**Implanted site**
Cartilage	Surface	Irregularity	Smooth	Smooth	Smooth
	Matrix	Fibrous tissue	Fibrocartilage	Fibrocartilage	Mixture; hyaline/fibrocartilage (transformation)
Subchondral bone		Granulation tissue	Increased remodeling	Detached (in the bottom half of the defect)	Increased remodeling (endochondral ossification)

Consistent with a previous study on rabbits [21], we report here the successful outcome of osteochondral regeneration with scaffold-free AT-MSC constructs in MMPigs. Although further studies will be required, we conclude that implantation of a scaffold-free 3D construct of AT-MSCs into an osteochondral defect can regenerate the original structure of the bone and cartilage.

## Conclusions

This pilot study suggests that implantation of a scaffold-free 3D construct of AT-MSCs into an osteochondral defect can induce regeneration of the original structure of the cartilage and subchondral bone over the course of 1 year.

## Additional files

Additional file 1:
**Surgical procedure, CT images, macroscopic findings of the articular surface, and histopathology of osteochondral defects in animal no. 3.** **Figure S1.** surgical procedure: A columnar construct (6 mm in diameter and 8 mm in height) composed of about 1,150 spheroids of AT-MSCs (A). An elliptic cylindrical osteochondral defect in each groove (B). Two constructs were autografted into the defect of the right hind limb (C). No implantation was in the left limb (B). **Figure S2.** CT images after the surgery: One cross section of the multi-planar reconstruction images 1, 6, and 12 months after the surgery in animal no. 3. In the implanted site, the radiopaque area gradually progressed and filled throughout the osteochondral defect after 12 months. However, in the control site, the spread of the radiopaque area was limited in the shallow layer, and no bone formation was in the deep layer. **Figure S3.** macroscopic findings of the articular surface: The surface was completely covered with abundant cartilaginous white tissues. The boundary to the surrounding normal cartilage was not different between the implanted site (A) and the control site (B). **Figure S4.** histopathology of osteochondral defects: At the implanted site, the restored subchondral bone was covered by mixture of hyaline/fibrocartilage, in which the clusters (arrowhead) and columnar clusters (arrow) of the cells were seen (A, B, C, D). In the control site, the surface was irregular, and the large fibrous tissue was presented in the subchondral (area with no bone at the bottom half of the defect (E, F, G, H)). Black dotted lines indicate the areas of osteochondral defects immediately after the surgery. Images B and F are high-power fields of the red dotted square in images A and E, respectively. The small images in sections B and F are high-power fields of white dotted squares in the respective images. The bars in the small images indicate 50 μm. Additional file 2:
**ICRS gross grading scale and histological grading scale in animal no. 3.** **Table S1.** ICRS gross grading scale. **Table S2.** ICRS histological grading scale.

## Abbreviations

AGG: Aggrecan
ALP: Alkaline phosphatase
AP2: Adipocyte fatty acid-binding protein 2
AT: Adipose tissue
AT-MSCs: Adipose tissue-derived mesenchymal stem cells
BM: Bone marrow
BM-MSCs: Bone marrow-derived mesenchymal stem cells
CCM: Complete culture medium
C-MYC: Cellular myelocytomatosis oncogene
Col-II: Collagen type II
CT: Computed tomography
DMEM: Dulbecco’s modified Eagle’s medium
EDTA: Ethylenediaminetetraacetate
FACS: Fluorescence-activated cell sorting
FBS: Fetal bovine serum
KLF-4: Krüppel-like factor 4
MFI: Mean fluorescence intensity
MMPigs: Microminipigs
MSCs: Mesenchymal stem cells
NANOG: Homeobox protein NANOG
NBF: Neutral buffered formalin
TGF-β3: Transforming growth factor beta 3
OA: Osteoarthritis
OC: Osteocalcin
OCT-4: Octamer-binding transcription factor 4
ON: Osteonectin
PBS: Phosphate-buffered saline
PPAR-γ2: Peroxisome proliferator-activated receptor γ2
SB: Staining buffer
SOX-2: Sex-determining region Y box 2
SOX-9: Sex-determining region Y-box 9
