# Supplementary material for: A preliminary study of osteochondral regeneration using a scaffold-free three-dimensional construct of porcine adipose tissue-derived mesenchymal stem cells
Source: J Orthop Surg Res. 2015 Mar 18;10:35. doi: 10.1186/s13018-015-0173-0 (PMC4389925; doi:10.1186/s13018-015-0173-0)

Additional File 1

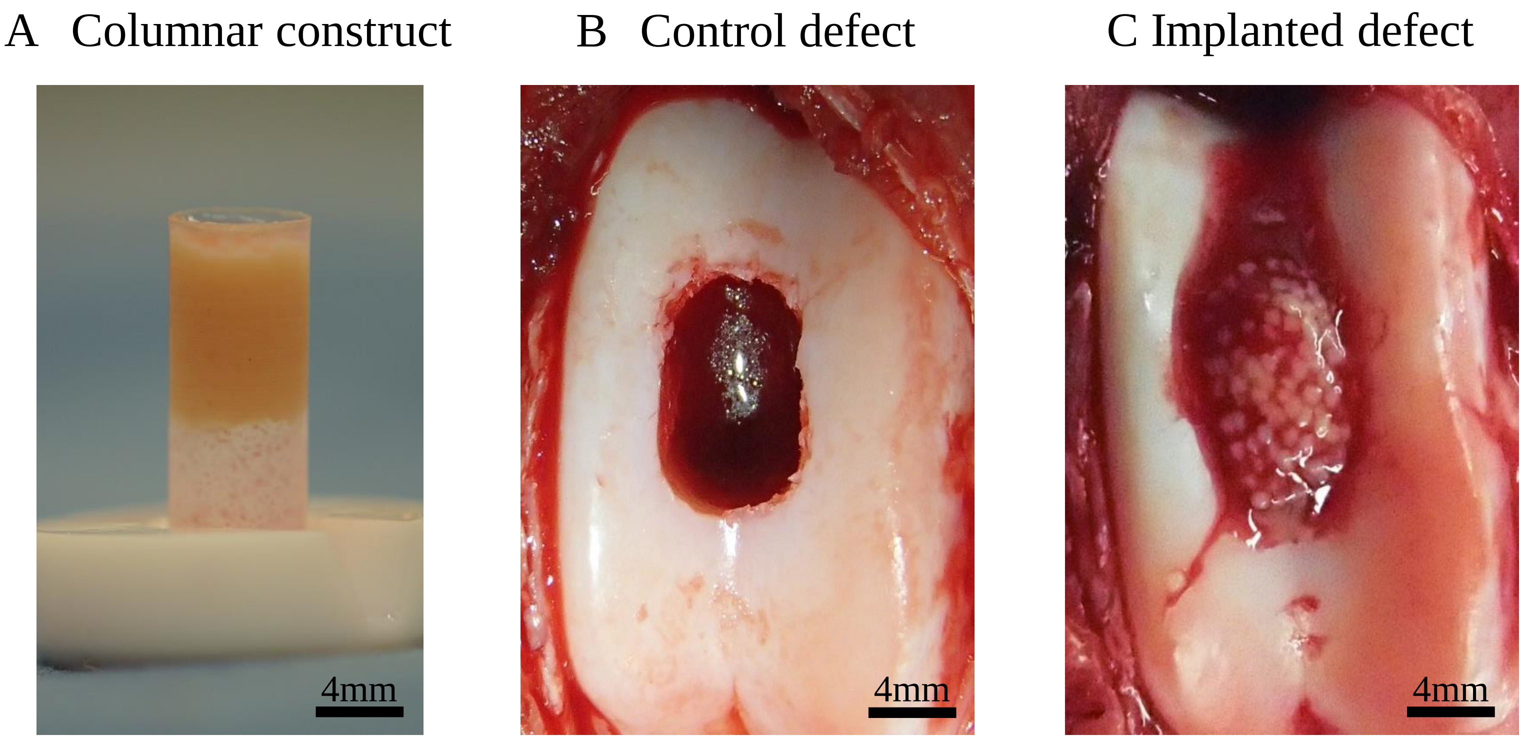

Surgical procedure in animal No.3.
A columnar construct (6 mm in diameter and 8 mm in height) composed of about 1150 spheroids of AT-MSCs (A). A elliptic cylindrical osteochondral defect in each groove (B). Two constructs were autografted into the defect of the right hind limb (C). No implantation was in the left limb (B).

Additional File 2

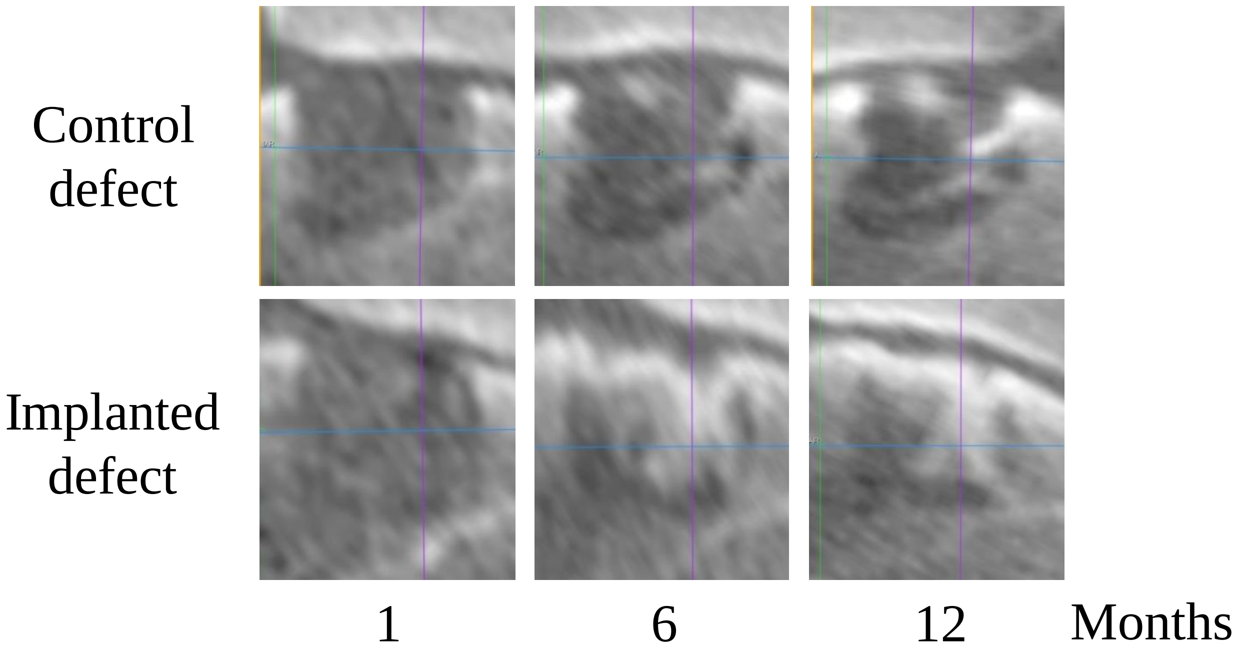

<u>CT images after the surgery in animal No.3.</u>

One cross-section of the multi-planar reconstruction images 1, 6, and 12 months after the surgery in animal No.3. In the implanted site, the radiopaque area gradually progressed, and filled throughout the osteochondral defect after 12 months. However, in the control site, the spread of radiopaque area was limited in the shallow layer, and no bone formation was in the deep layer.

Additional File 3

A Implanted defect B Control defect

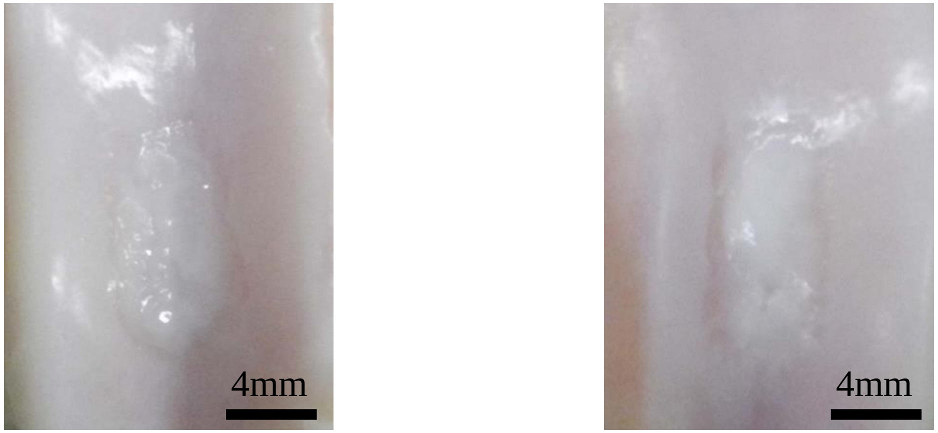

<u>Macroscopic findings of the articular surface in animal No.3</u>

The surface was completely covered with abundant cartilageous white tissues.
The boundary to the surrounding normal cartilage was not different between the implanted site (A) and the control site (B).

Additional File 5

<u>Histopathology of osteochondral defects of animal No.3</u>

At the implanted site, the restored subchondral bone was covered by mixture of hyaline/fibrocartilage, in which the clusters (arrowhead) and columnar clusters (arrow) of the cells were seen (A, B, C, D). In the control site, the surface was irregular, and the large fibrous tissue was presented in the subchondral (area no bone in bottom half of the defect (E, F, G, H). Black dotted lines indicate the areas of osteochondral defects immediately after the surgery. The images B and F are high power fields of red dotted square in the images A and E , respectively. The small images in the sections B and F were high power fields of white dotted squares in the respective images. The bars in the small images indicate 50 μm.

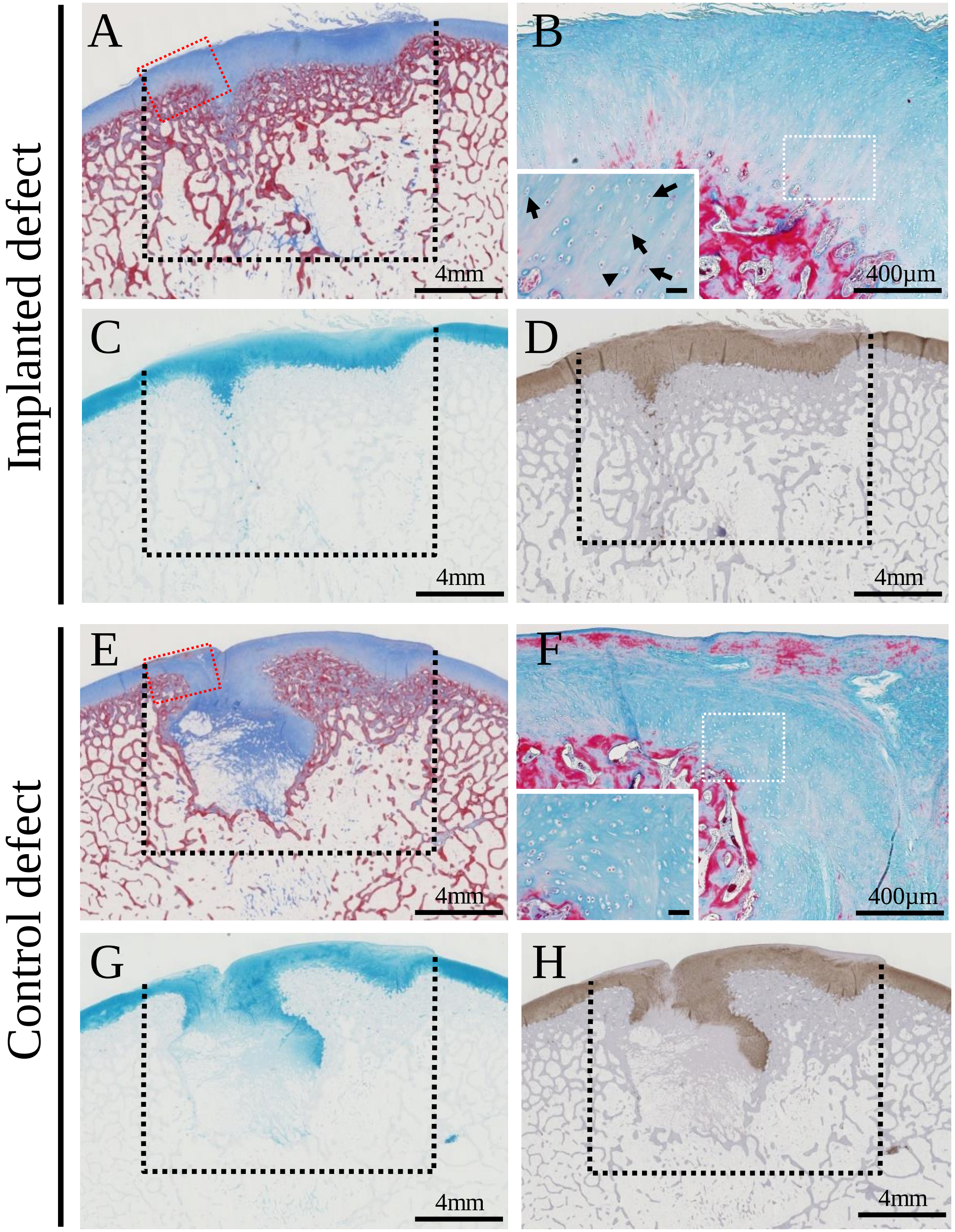

Supplement: Additional file 1: — Surgical procedure, CT images, macroscopic findings of the articular surface, and histopathology of osteochondral defects in animal no. 3. Figure S1. surgical procedure: A columnar construct (6 mm in diameter and 8 mm in height) composed of about 1,150 spheroids of AT-MSCs (A). An elliptic cylindrical osteochondral defect in each groove (B). Two constructs were autografted into the defect of the right hind limb (C). No implantation was in the left limb (B). Figure S2. CT images after the surgery: One cross section of the multi-planar reconstruction images 1, 6, and 12 months after the surgery in animal no. 3. In the implanted site, the radiopaque area gradually progressed and filled throughout the osteochondral defect after 12 months. However, in the control site, the spread of the radiopaque area was limited in the shallow layer, and no bone formation was in the deep layer. Figure S3. macroscopic findings of the articular surface: The surface was completely covered with abundant cartilaginous white tissues. The boundary to the surrounding normal cartilage was not different between the implanted site (A) and the control site (B). Figure S4. histopathology of osteochondral defects: At the implanted site, the restored subchondral bone was covered by mixture of hyaline/fibrocartilage, in which the clusters (arrowhead) and columnar clusters (arrow) of the cells were seen (A, B, C, D). In the control site, the surface was irregular, and the large fibrous tissue was presented in the subchondral (area with no bone at the bottom half of the defect (E, F, G, H)). Black dotted lines indicate the areas of osteochondral defects immediately after the surgery. Images B and F are high-power fields of the red dotted square in images A and E, respectively. The small images in sections B and F are high-power fields of white dotted squares in the respective images. The bars in the small images indicate 50 μm. [file 13018_2015_173_MOESM1_ESM.pdf]
